# The Vascularity of Tissue

**Published:** 1889-05

**Authors:** 


### THE VASCULARITY OF TISSUE.

"The term vascular we use as it is found in the text-books. We all admit that there is a lymphatic circulation, as, for instance, in the cornea of the eye; but when we speak of an inflammation in non-vascular tissues, we confine ourselves to the generally used terms. True inflammation is always connected with a vascular supply. Red and white blood corpuscles do not penetrate into the normal cornea. It is only after the lymphatics are dilated that the red and white corpuscles can penetrate, and we then have true inflammation set up. The same applies to the dentine, and as the tubes are not dilatable, blood corpuscles cannot enter, so can no true inflammation be set up."

"The only living tissues found in the dentine are the dentinal fibrils, and they are processes of the odontoblasts, and consequently derive their support from the parent cells, which lie in direct connection with the vascular supply. Consequently, it is not necessary to account for any circulation in the dentine except that produced by osmosis, and that we fully accept and have always held."

"There is no question in the minds of working histologists and pathologists regarding the part played by the tooth in the process of decay. The formed material of the tooth is a perfectly passive agent in the retrograde process. The only resistance expressed as the result of irritation to the dentinal fibrils is found in the pulp-cavity, where the persistent odontoblasts lie upon the surface of the pulp, and we call the product secondary dentine."

